# Comparison of the efficacy of intravitreal triamcinolone acetonide for cystoid macular edema with versus without serous retinal detachment in branch retinal vein occlusion: influence on macular sensitivity and morphology

**DOI:** 10.1186/1471-2415-12-39

**Published:** 2012-08-09

**Authors:** Hidetaka Noma, Hideharu Funatsu, Tatsuya Mimura, Katsunori Shimada

**Affiliations:** 1 Department of Ophthalmology, Yachiyo Medical Center, Tokyo Women’s Medical University, 477-96, Owada-shinden, Yachiyo, Chiba 276-8524, Japan; 2 Department of Ophthalmology, University of Tokyo Graduate School of Medicine, Tokyo, Japan; 3 Department of Hygiene and Public Health II, Tokyo Women’s Medical University, Tokyo, Japan

## Abstract

**Background:**

The influence of serous retinal detachment (SRD) on visual acuity, macular sensitivity, and macular thickness is unclear after intravitreal injection of triamcinolone acetonide (IVTA) for macular edema with branch retinal vein occlusion (BRVO).

**Methods:**

Twenty-one eyes of 21 BRVO patients with macular edema received IVTA. Patients were divided into two groups by optical coherence tomography findings: 11 patients who had cystoid macular edema (CME) with SRD (SRD (+) group) and 10 patients who had CME without SRD (SRD (−) group). Microperimetry was performed with a Micro Perimeter 1 before and at 3 and 6 months after IVTA. Macular thickness was measured by optical coherence tomography. We exchanged the superior and inferior regions to separate the regions into those with and without occlusion. As a result, the superior region was always the occluded region and the inferior region was non-occluded.

**Results:**

In both the SRD (−) group and the SRD (+) group, the mean macular thickness within the central 4° field and the 10° and 20° fields of the occluded region decreased significantly from baseline to 3 and 6 months after IVTA (all P <0.01). Visual acuity also improved significantly in both groups from baseline to 3 and 6 months after IVTA (both P <0.05). In both groups, the mean macular sensitivity (measured with by microperimetry) within the central 4° field and the 10° and 20° fields of the occluded region showed a significant increase from baseline to 3 and 6 months after IVTA (all P <0.05). The trend profiles of macular thickness within the 10° and 20° fields of the occluded region showed significant differences, but there were no significant differences with respect to the trend profiles of visual acuity and macular sensitivity within the central 4° field and the 10° and 20° fields of the occluded region.

**Conclusions:**

These results suggest that IVTA may achieve more marked improvement of macular morphology in BRVO patients with SRD than in those without SRD, while this therapy may have a similar effect on macular function in BRVO patients with or without SRD.

## Background

Branch retinal vein occlusion (BRVO) is a common retinal vascular disease that often leads to macular edema, which is the chief reason for visual impairment in BRVO patients [1,2]. An increase of pressure and reduction of blood flow in the macular capillaries can lead to dysfunction of the endothelial blood-retinal barrier and an increase of vascular permeability that results in macular edema [3]. Recent randomized, controlled clinical trials have evaluated several treatment modalities, including intravitreal triamcinolone acetonide [4] and anti-vascular endothelial growth factor (VEGF) therapy [5] for macular edema in patients with BRVO, and both treatments have been reported to improve visual acuity after 12 months. We previously reported that VEGF and inflammatory factors may contribute to the pathogenesis of macular edema associated with BRVO [6-9], which provides a rationale supporting the efficacy of intravitreal triamcinolone (IVTA) and anti-VEGF therapy. However, previous clinical studies only employed measurement of visual acuity to evaluate visual function, even though macular edema usually involves the larger macular area and not just the fovea. The Micro Perimeter 1 (MP-1) is an instrument that combines digital fundus imaging with automated perimetry [10,11]. Unlike measurement of visual acuity that only reflects foveal function, the MP-1 can evaluate both the fovea and the larger macular area. We have previously found that retinal thickness and retinal volume are more closely related to retinal sensitivity than to visual acuity in BRVO patients who have macular edema [12].

Optical coherence tomography (OCT) has revealed that macular edema secondary to BRVO is frequently associated with serous retinal detachment (SRD), as well as with cystoid macular edema (CME) and inner retinal thickening [13-15]. Some authors have reported that the visual prognosis is poor for BRVO patients with SRD [14,16-18]. It has been reported that the retinal thickness is greater in SRD patients than CME patients [14], and that IVTA decreases retinal thickness in SRD patients [16]. Therefore, it may be important to investigate differences between SRD and CME. In addition, our previous cross-sectional study showed that visual acuity and macular thickness within the central 4°, 10°, and 20° fields were significantly worse in the SRD group than in the CME group, while macular sensitivity within the central 4°, 10°, and 20° fields did not differ significantly between the two groups [19]. However, little is known about the influence of SRD in BRVO patients receiving IVTA for macular edema. Therefore, we performed the present study to assess the influence of SRD on changes of visual acuity, macular sensitivity, and macular thickness after IVTA in BRVO patients with macular edema.

## Methods

### Subjects

This study was approved by the Institutional Ethics Committee of Tokyo Women’s Medical University and adhered to the tenets of the Declaration of Helsinki. Written informed consent was obtained from each patient. IVTA was performed as part of standard care because it has been reported that macular edema and visual acuity can be improved in BRVO patients by this procedure [4]. At our hospital, the treatment options for macular edema associated with BRVO include follow-up, intravitreal anti-VEGF therapy, IVTA, laser photocoagulation, and pars plana vitrectomy. This study enrolled the patients in whom IVTA was selected as the management option for CME, including SRD. We prospectively studied 21 eyes of 21 consecutive patients (mean age: 69.4 ± 9.6 years; 14 women and 7 men) who had BRVO with macular edema and were treated with IVTA. This prospective uncontrolled study was conducted at the Department of Ophthalmology of Tokyo Women’s Medical University between August 2008 and March 2011. Patients were diagnosed as having hypertension if the systolic blood pressure was ≥140 mm Hg and diastolic blood pressure was >90 mm Hg, or if the systolic pressure was ≥140 mm Hg at one examination and the diastolic pressure was ≥90 mm Hg on a different day, or if the patient was already taking antihypertensive medication [20]. A diagnosis of hyperlipidemia was based on a total cholesterol≥240 mg/dL, triglycerides≥160 mg/dL, low-density lipoprotein cholesterol≥130 mg/dL, or use of cholesterol-lowering medication [20].

The inclusion criteria were eyes with a foveal thickness greater than 300 μm and a visual acuity equal to or less than 20/30. The exclusion criteria were (1) previous ocular surgery, (2) diabetes mellitus with diabetic retinopathy, (3) previous macular laser photocoagulation, (4) previous intravitreal injection of anti-VEGF agents or triamcinolone acetonide, (5) a history of ocular inflammation, (6) marked retinal hemorrhage (including macular bleeding involving the intrafoveal or subfoveal spaces), (7) coexisting ocular disease (i.e., epiretinal membrane or glaucoma), and (8) retreatment during the 6-month follow-up period. Twelve patients had superior vein occlusion and 9 patients had inferior occlusion.

All patients had undergone a comprehensive ophthalmologic examination, including best-corrected visual acuity measurement, intraocular pressure determination, indirect ophthalmoscopy, and slit-lamp biomicroscopy with a contact lens before and at 3, and 6 months after treatment. In addition, retinal sensitivity was investigated by microperimetry, and retinal thickness was measured by OCT.

### Surgical procedure

In the current study, 21 patients received IVTA under local anaesthesia. For intravitreal therapy, the most common dosage of triamcinolone acetonide was 4.0 mg in a volume of 0.1 ml. Injection of triamcinolone into the vitreous fluid was done via the pars plana at 3–4 mm posterior to the limbus. All injections were performed with a sterile technique, and prophylactic topical antibiotics were applied for 1 week after injection. All patients were followed up for at least 6 months postoperatively. Recurrence of macular edema was defined as an increase (by >100 μm compared with the value at 3 months after initial IVTA) of foveal thickness when the thickness had once decreased to <300 μm at 3 months after initial IVTA [11]. Recurrence of macular edema was observed in 3/21 eyes (14%), but the 3 patients did not want further treatment.

### Fundus examination

As baseline screening, patients underwent ophthalmoscopy and biomicroscopic examination using a slit-lamp with a fundus contact lens. They also underwent standard fundus color photography and fluorescein angiography, which was performed with a Topcon TRC-50EX fundus camera, an image-net system (Tokyo Optical Co. Ltd., Japan), and a preset lens with a slit-lamp.

A masked grader independently assessed ischemic retinal vascular occlusion on the fluorescein angiograms by measuring the ischemic area of the retina with the public domain Scion Image program, as reported previously [6-8]. On digital photographs of the fundus, the optic disc was outlined with a cursor and then its area was measured, as was also done for the nonperfused area of the retina. Then the nonperfused area was divided by the disc area to calculate the severity of retinal ischemia.

### Measurement of BCVA

Each patient underwent measurement of best-corrected visual acuity (BCVA) with an SC-2000 System chart (Nidek, Gamagori, Japan). BCVA was measured in decimal units on a Landolt chart by the orthoptists. The chart brightness was set at 80–320 cd/m^2^, and chart contrast was more than 74%. The results were converted to the logarithm of the minimum angle of resolution scale (log MAR).

### Measurement of optical coherence tomography

OCT was performed with an instrument from Zeiss-Humphrey Ophthalmic Systems (Zeiss Stratus OCT3, Carl Zeiss Meditec, Dublin, CA, USA) to measure the foveal thickness. At each visit, all patients underwent Stratus OCT examination in the vertical cross-section with the instrument centered on the fovea and in the fast macular thickness mode. On these views, retinal thickness was defined as the distance between the inner surface of the neurosensory retina and the retinal pigment epithelium. Foveal thickness was calculated as the average retinal thickness within a circle of 500-μm radius centered on the fovea. A retinal thickness map and retinal volume map were obtained by scanning 6×6 mm (20°×20°) areas of the macular region, which was divided into the following nine subfields: 1) fovea, 2) superior inner macula, 3) nasal inner macula, 4) inferior inner macula, 5) temporal inner macula, 6) superior outer macula, 7) nasal outer macula, 8) inferior outer macula, and 9) temporal outer macula [12]. The diameters of the central, inner, and outer circles were 1, 3, and 6 mm, respectively. In each region, measurement of retinal thickness was automatically performed by computer software.

In this study, we exchanged the superior and inferior regions to separate the regions into those with occlusion and those without it. As a result, the superior region was always the occluded region and the inferior region was always non-occluded. We then analyzed the occluded and non-occluded regions separately. For the occluded region, the mean macular thickness was determined across four subfields (fovea, superior inner, nasal inner, and temporal inner) covering the central 3×3 mm (10°×10°), as well as across seven subfields (fovea, superior inner, nasal inner, temporal inner, superior outer, nasal outer, and temporal outer) covering the central 6×6 mm (20°×20°). For the non-occluded region, the mean macular thickness was determined in one subfield (inferior inner) covering the central 3×3 mm (10°×10°), as well as across two subfields (inferior inner and inferior outer) covering the central 6×6 mm (20°×20°). The mean macular thickness was also determined in one subfield (fovea) covering the central 1×1 mm (4°×4°).

SRD was defined as being present if OCT revealed typical accumulation of subretinal fluid resulting in neurosensory retinal detachment with low or absent reflectivity anterior to a clearly distinguishable outer band, irrespective of the coexistence of CME [21]. CME was defined as present if OCT revealed hyporeflective intraretinal cavities. All 11 patients with SRD had both SRD and CME. Therefore, we classified the subjects into a CME without SRD group (SRD (−) group) and a CME with SRD group (SRD (+) group).

### Functional mapping by microperimetry

Microperimetry with the MP-1 (Nidek, Gamagori, Japan) is performed using an infrared fundus camera with a liquid crystal display controlled by special software. The MP-1 software contains an automatic tracking system for fundus movements; this evaluates every acquired frame for shifts in the x and y directions of the fundus with respect to a reference frame obtained by an infrared camera at the beginning of the examination. Each patient underwent fundus-monitored microperimetry with the MP-1 system (Nidek, Gamagori, Japan). Its software performs automatic tracking of fundus movements and evaluates every frame acquired for fundus shift in the x and y directions relative to a reference frame obtained with an infrared camera at the beginning of the examination. Microperimetry settings were identical for all examinations: Goldmann III stimuli were presented in random order according to a 4-2-1 double staircase strategy. The stimulus intensity ranged from 0 to 20 decibels (dB) (0 dB corresponded to the strongest signal intensity of 127 cd/m^2^) in 1-dB steps, and the duration of each stimulus was 200 ms. The fixation target was varied in size according to the patient's visual acuity. Retinal sensitivity maps were obtained by using the macula 20 degrees program of the MP-1. During the examination, background illumination was set at 1.27 cd/m^2^. Mean retinal sensitivity was calculated from the sensitivity for each of nine subfields on the retinal map generated by OCT. We analyzed data for the occluded and non-occluded regions separately. For the occluded region, we determined the mean macular sensitivity at 23 sites in the central 10° field (four subfields: fovea, superior inner, nasal inner, and temporal inner) and at 44 sites in the central 20° field (seven subfields: fovea, superior inner, nasal inner, temporal inner, superior outer, nasal outer, and temporal outer). For the non-occluded region, we determined the mean macular sensitivity at 6 sites in the central 10° field (inferior inner subfield) and at 13 sites in the central 20° field (inferior inner and inferior outer subfields). The mean macular sensitivity was also determined at five sites in the central 4° field.

### Statistical analysis

All analyses were performed with SAS System 9.1 software (SAS Institute Inc., Cary, North Carolina, USA). Results are presented as the mean ± SD or as the frequency. One-way or two-way repeated measures ANOVA was used to evaluate changes in visual acuity, macular sensitivity, and macular thickness. To examine differences in the changes of parameters over time, trend profiles were determined by repeated measures analysis. Two-tailed P values of less than 0.05 were considered to indicate statistical significance.

## Results

The characteristics of the SRD (−) group and the SRD (+) group are summarized in Table 1. Among the 21 patients with BRVO, 10 were assigned to the SRD (−) group and 11 to the SRD (+) group. The mean age, female/male ratio, prevalence of hypertension, prevalence of hyperlipidemia, duration of BRVO, pattern of BRVO, and nonperfused area were similar in the SRD (−) group and the SRD (+) group (*P* = 0.448, *P* = 0.537, *P* = 0.890 *P* = 0.801, *P* = 0.287, *P* = 0.890, and *P* = 0.932, respectively).

**Table 1:** Baseline clinical features of the two groups

**Findings**	**SRD (-) (N = 10)**	**SRD (+)(N = 11)**	**P value**
Age (years)	71.2±7.3^‡^	67.9±11.4^‡^	0.448
Gender (female/male)	6/4	8/3	0.537
Hypertension	7	8	0.890
Systolic blood pressure (mmHg)	134±13^‡^	137±15^‡^	0.535
Diastolic blood pressure (mmHg)	83±8^‡^	84±16^‡^	0.806
Hyperlipidemia	6	6	0.801
Duration of BRVO (months)	4.8±3.0^‡^	3.6±1.7^‡^	0.287
Pattern of BRVO (major/macular)	7/3	8/3	0.890
Nonperfused area (disc areas)	39.0±43.8^‡^	37.5±32.6^‡^	0.932

BRVO = branch retinal vein occlusion; SRD = serous retinal detachment. ^‡^Mean ± standard deviation (SD).

**Figure 1:**
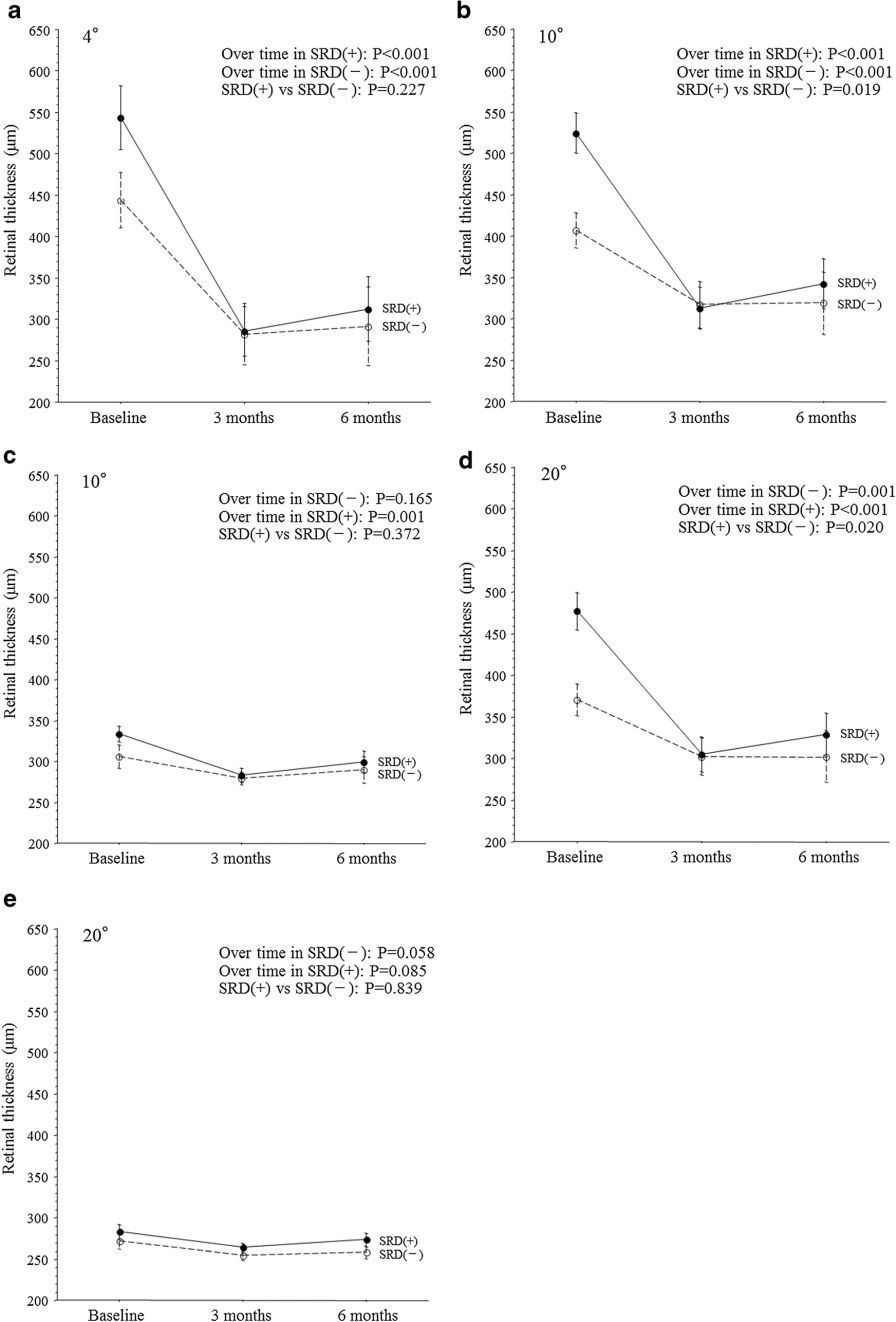
**Trend profiles of the mean macular thickness after intravitreal injection of triamcinolone acetonide (IVTA) in BRVO patients with macular edema. **(**a**) Central 4° field (SRD (+), P<0.001; SRD (-), P<0.001; SRD (+) vs. SRD (-), P=0.227). (**b**) 10° field of the occluded region (SRD (+), P<0.001; SRD (-), P<0.001; SRD (+) vs. SRD (-), P=0.019). (**c**) 10° field of the non-occluded region (SRD (+), P=0.001; SRD (-), P=0.165; SRD (+) vs. SRD (-), P=0.372). (**d**) 20° field of the occluded region (SRD (+), P<0.001; SRD (-), P=0.001; SRD (+) vs. SRD (-), P=0.020). (**e**) 20° field of the non-occluded region (SRD (+), P=0.085; SRD (-), P=0.058; SRD (+) vs. SRD (-), P=0.839).

In both groups, IVTA achieved a reduction of clinical macular edema that was confirmed by OCT. In both groups, the mean macular thickness within the central 4° field decreased significantly from baseline to 3 and 6 months after IVTA (Figure 1a). In both groups, the mean macular thickness within the 10° field of the occluded region also decreased significantly from baseline to 3 and 6 months after IVTA (Figure 1b). In the SRD (−) group, the mean macular thickness within the 10° field of the non-occluded region did not decrease significantly from baseline to 3 and 6 months after IVTA (Figure 1c). On the other hand, the mean macular thickness within the 10° field of the non-occluded region decreased significantly from baseline to 3 and 6 months in the SRD (+) group (Figure 1c). In both groups, the mean macular thickness within the 20° field of the occluded region showed a significant decrease from baseline to 3 and 6 months after IVTA (Figure 1d). However, the mean macular thickness within the 20° field of the non-occluded region did not decrease significantly from baseline to 3 and 6 months in either group (Figure 1e).

**Figure 2:**
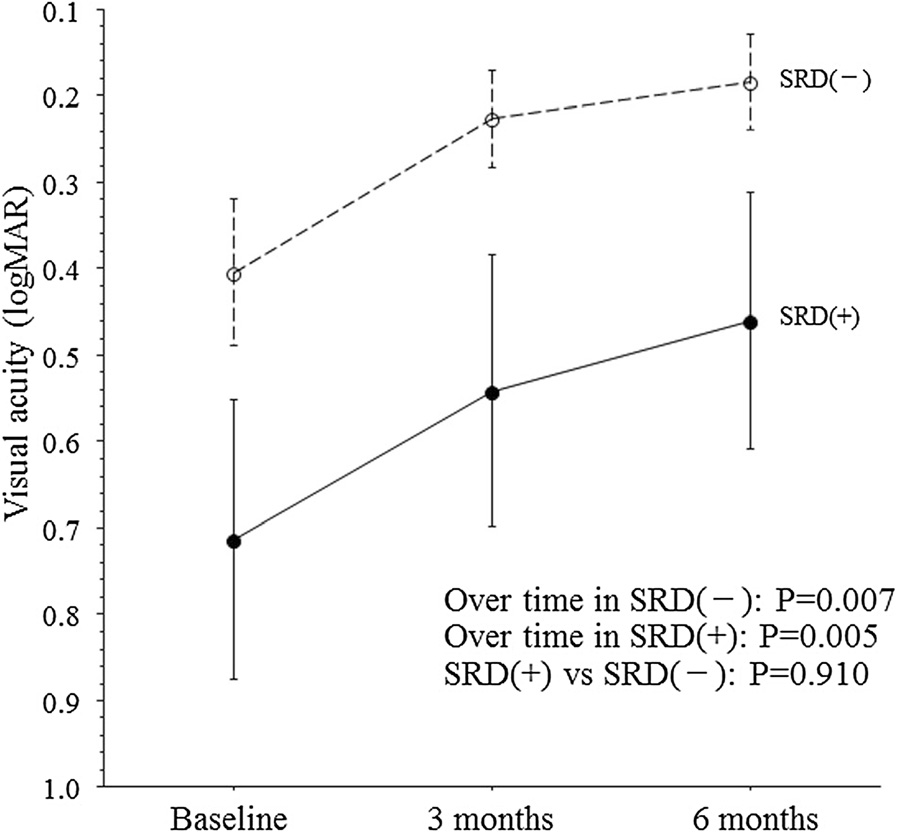
**Trend profile of the mean visual acuity after intravitreal injection of triamcinolone acetonide (IVTA) in BRVO patients with macular edema. **SRD (+), P=0.005; SRD (-), P=0.007; SRD (+) vs. SRD (-), P=0.910.

In both groups, postoperative improvement of visual acuity was noted after IVTA and persisted for at least 6 months. The visual acuity of the SRD (−) group showed significant improvement from baseline to 3 and 6 months after IVTA (Figure 2). Likewise, the visual acuity of the SRD (+) group showed significant improvement from baseline to 3 and 6 months after IVTA (Figure 2).

**Figure 3:**
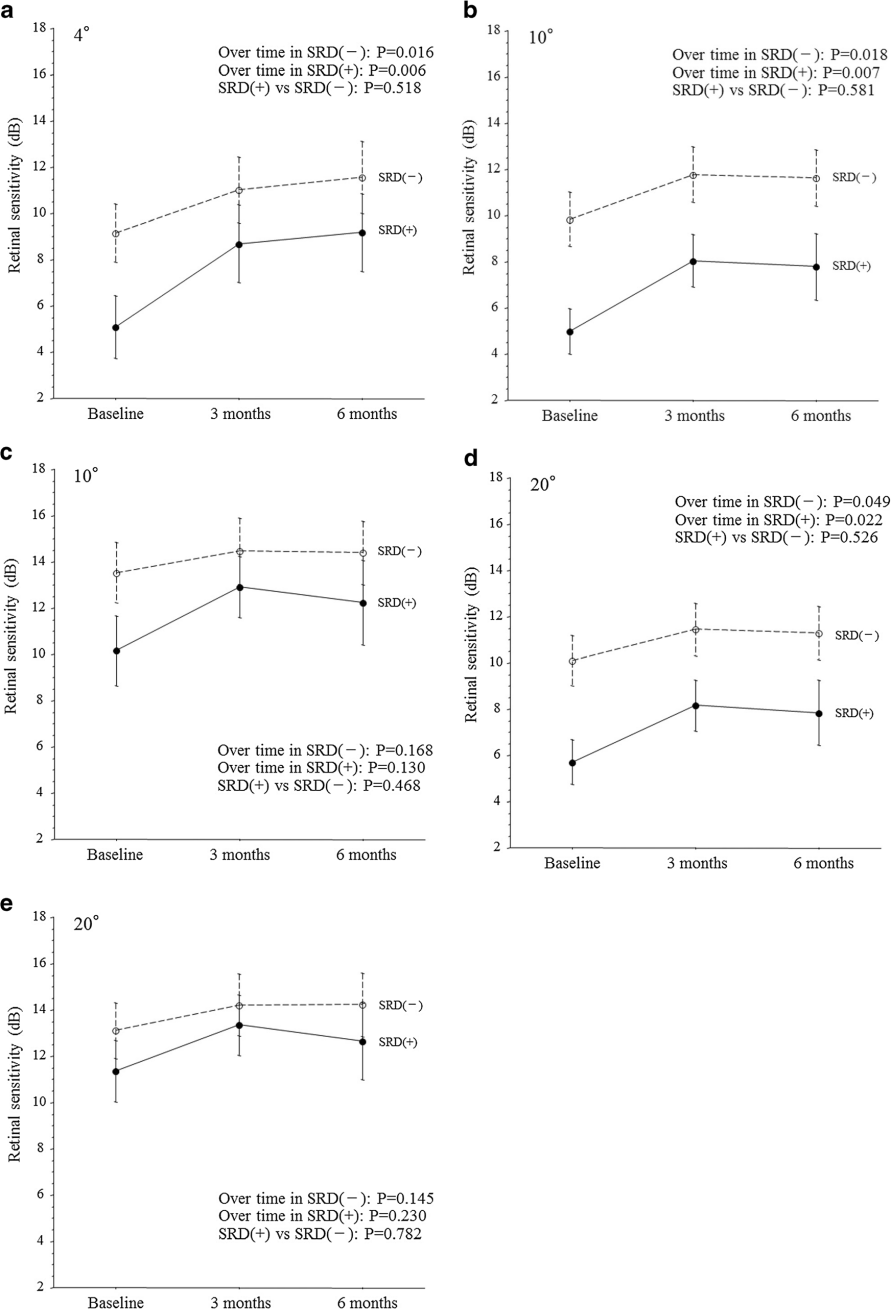
**Trend profiles of the mean macular sensitivity after intravitreal injection of triamcinolone acetonide (IVTA) in BRVO patients with macular edema. **(**a**) Central 4° field (SRD (+), P=0.006; SRD (-), P=0.016; SRD (+) vs. SRD (-), P=0.518). (**b**) 10° field of the occluded region (SRD (+), P=0.007; SRD (-), P=0.018; SRD (+) vs. SRD (-), P=0.581). (**c**) 10° field of the non-occluded region (SRD (+), P=0.130; SRD (-), P=0.168; SRD (+) vs. SRD (-), P=0.468). (**d**) 20° field of the occluded region (SRD (+), P=0.022; SRD (-), P=0.049; SRD (+) vs. SRD (-), P=0.526). (**e**) 20° field of the non-occluded region (SRD (+), P=0.230; SRD (-), P=0.145; SRD (+) vs. SRD (-), P=0.782).

In both the SRD (−) group and the SRD (+) group, improvement of macular function after IVTA was detected with the MP-1. In both groups, the mean macular sensitivity within the central 4° field increased significantly from baseline to 3 and 6 months after IVTA (Figure 3a). In both groups, the mean macular sensitivity within the 10° field of the occluded region also showed a significant increase from baseline to 3 and 6 months after IVTA (Figure 3b). However, the mean macular sensitivity within the 10° field of the non-occluded region did not show a significant increase from baseline to 3 and 6 months after IVTA in either group (Figure 3c). In both groups, the mean macular sensitivity within the 20° field of the occluded region increased significantly from baseline to 3 and 6 months after IVTA (Figure 3d). However, the mean macular sensitivity within the 20° field of the non-occluded region did not show a significant increase from baseline to 3 and 6 months after IVTA (Figure 3e).

There were no significant differences between the SRD (−) group and the SRD (+) group with regard to the trend profiles of macular thickness within the central 4° field or within the 10° and 20° fields of the non-occluded region (Figures 1a, c, and e). On the other hand, there were significant differences between the two groups with respect to the trend profiles of macular thickness within the 10° and 20° fields of the occluded region (Figures 1b and d). The trend profile of visual acuity showed no significant difference between the two groups (Figure 2). There were also no significant between-group differences with regard to the trend profiles of macular sensitivity within the central 4° field or within the 10° and 20° fields of the occluded and non-occluded regions (Figures 3a-e).

Up to 6 months after IVTA, two of the 21 patients (9.5%) showed an increase of intraocular pressure, but this could be controlled by a change of medication. Neovascular glaucoma was not detected in any of the patients, so trabeculectomy or shunt surgery was not performed. There were also no new cases of cataract or progression of existing cataracts and no infectious endophthalmitis after IVTA.

## Discussion

In the present study, we found that the mean macular thickness within the central 4° field and within the 10° and 20° fields of the occluded region showed a significant decrease from baseline to 3 and 6 months after IVTA in both the SRD (−) group and the SRD (+) group. These results suggest that macular thickness is improved by IVTA in BRVO patients with SRD, as has already been demonstrated in BRVO patients without SRD. SRD is thought to be caused by transudation of extracellular fluid into the subretinal space [13-15,22,23], with the site of detachment being determined by the foveal architecture, especially the Müller cell cone [14]. When the barrier function of the external limiting membrane (ELM) is lost due to traction on the Müller cell cone, intraretinal fluid will move into the subretinal space, resulting in an increase of SRD and alleviation of retinal edema [14]. Triamcinolone acetonide is thought to improve macular edema by decreasing retinal capillary permeability via an effect on tight junctions [24], or it could inhibit the signaling cascade involving VEGF and the VEGF receptor that increases microvascular permeability [25,26]. Corticosteroids may also prevent the production of various inflammatory molecules [27-29] that promote leukocyte adhesion and breakdown of the blood-retinal barrier [30,31]. We previously reported that an excessive increase of vascular permeability secondary to upregulation of VEGF and sICAM-1 may contribute to the development of SRD in BRVO patients [21]. Accordingly, a decrease of retinal capillary permeability after IVTA may relieve traction on the Müller cell cone, resulting in a decrease of SRD. Thus, IVTA may be effective for reducing macular thickness in BRVO patients with SRD as well as those without SRD.

We also found that visual acuity and macular sensitivity within the central 4° field and within the 10°, and 20° fields of the occluded region were improved significantly from baseline to 3 and 6 months after IVTA in both the SRD (−) group and the SRD (+) group. These results suggest that visual acuity and macular sensitivity can also be improved by IVTA in BRVO patients with SRD as has been shown in BRVO patients without SRD. In eyes with BRVO, fluid leaking from affected retinal capillaries accumulates around the fovea and produces retinal thickening, which may cause the internal limiting membrane at the clivus of the fovea to protrude with formation of foveal cystoid spaces [14]. The cells in the Müller cell cone would then be stretched perpendicularly in the walls of the foveal cystoid spaces. When fluid leakage increases, further traction on the Müller cell cone would lead to traction on the inner and outer segments of the foveal photoreceptors, resulting in a small retinal detachment at the fovea [14]. Subsequently, SRD would develop when the ELM barrier breaks down at the fovea [14]. Loss of the ELM barrier often leads to damage to photoreceptors in the outer segment, resulting in impairment of macular function (visual acuity and macular sensitivity). These reports and our results suggest that reduced traction on the Müller cell cone due to a decrease of vascular permeability after IVTA can alleviate damage to foveal photoreceptors in the outer segment by restoring the ELM barrier, resulting in improvement of macular function (visual acuity and macular sensitivity) in BRVO patients whether or not they have SRD.

Interestingly, this study demonstrated a significant difference in the trend profiles of macular thickness within the 10° and 20° fields of the occluded region between the SRD (−) group and the SRD (+) group, while there were no significant differences between the two groups with regard to the trend profiles of visual acuity and macular sensitivity within the central 4° field and within the 10° and 20° fields of the occluded and non-occluded regions. In other words, macular thickness within the 10° and 20° fields showed a better response to IVTA in BRVO patients with SRD than in those without SRD. The greater improvement of morphology in the SRD (+) group compared with the SRD (−) group may have been due to the fact that SRD causes more extensive morphologic change compared with CME, thus allowing a stronger response to IVTA. On the other hand, visual acuity and macular sensitivity showed similar trend profiles after IVTA regardless of the presence or absence of SRD. This discrepancy between the trend profiles of macular thickness versus visual acuity and macular sensitivity may have arisen because SRD did not have much influence on macular function since it is caused by transudation of extracellular fluid into the subretinal space [13-15] and there is little traction on the Müller cell cone in the macular region [14]. Thus, our results suggest that IVTA may achieve more improvement of macular morphology in BRVO patients with SRD than in those without SRD, while this therapy may have a similar influence on macular function in BRVO patients with or without SRD. However, further investigation will be needed to clarify the relation between the changes of macular function and morphology after IVTA.

The limitations of this study were a short follow-up period, a small sample size, and lack of a control group. Although the results of long-term follow up could be interesting, additional treatment with further IVTA or anti-VEGF agents for recurrence may bias the outcome. Despite this risk, further investigations should be done to evaluate the long-term outcome in the future. In addition, we could not evaluate the relation between macular function and the inner segment/outer segment ratio of the photoreceptor layer because we did not have access to spectral domain OCT.

## Conclusions

There was a significant difference between the SRD (−) group and the SRD (+) group with respect to the trend profiles of macular thickness within the 10° and 20° fields of the occluded region, but there were no significant between-group differences with regard to the trend profiles of visual acuity and macular sensitivity within the central 4° field and within the 10°, and 20° fields of the occluded and non-occluded regions. These results suggest that IVTA may achieve more marked improvement of macular morphology in BRVO patients with SRD than in those without SRD, while this therapy may have a similar effect on macular function irrespective of the presence of SRD.

## Competing interests

No conflicting relationship exists for any author.

## Authors’ contributions

HN and HF were involved in the design and conduct of the study. Collection and management of the data were done by HN and KS while analysis and interpretation of the data were performed by HN HF TM and KS Preparation of the first draft of the manuscript was done by HN and review and approval of the manuscript was performed by HF and TM All authors read and approved the final manuscript.

## Financial support

None.

## Pre-publication history

The pre-publication history for this paper can be accessed here:

http://www.biomedcentral.com/1471-2415/12/39/prepub
